# Proteomics Analysis Reveals Previously Uncharacterized Virulence Factors in *Vibrio proteolyticus*

**DOI:** 10.1128/mBio.01077-16

**Published:** 2016-07-26

**Authors:** Ann Ray, Lisa N. Kinch, Marcela de Souza Santos, Nick V. Grishin, Kim Orth, Dor Salomon

**Affiliations:** a Department of Molecular Biology, University of Texas Southwestern Medical Center, Dallas, Texas, USA; b Howard Hughes Medical Institute, University of Texas Southwestern Medical Center, Dallas, Texas, USA; c Department of Biochemistry, University of Texas Southwestern Medical Center, Dallas, Texas, USA; d Department of Biophysics, University of Texas Southwestern Medical Center, Dallas, Texas, USA

## Abstract

Members of the genus *Vibrio* include many pathogens of humans and marine animals that share genetic information via horizontal gene transfer. Hence, the *Vibrio* pan-genome carries the potential to establish new pathogenic strains by sharing virulence determinants, many of which have yet to be characterized. Here, we investigated the virulence properties of *Vibrio proteolyticus*, a Gram-negative marine bacterium previously identified as part of the *Vibrio* consortium isolated from diseased corals. We found that *V. proteolyticus* causes actin cytoskeleton rearrangements followed by cell lysis in HeLa cells in a contact-independent manner. In search of the responsible virulence factor involved, we determined the *V. proteolyticus* secretome. This proteomics approach revealed various putative virulence factors, including active type VI secretion systems and effectors with virulence toxin domains; however, these type VI secretion systems were not responsible for the observed cytotoxic effects. Further examination of the *V. proteolyticus* secretome led us to hypothesize and subsequently demonstrate that a secreted hemolysin, belonging to a previously uncharacterized clan of the leukocidin superfamily, was the toxin responsible for the *V. proteolyticus*-mediated cytotoxicity in both HeLa cells and macrophages. Clearly, there remains an armory of yet-to-be-discovered virulence factors in the *Vibrio* pan-genome that will undoubtedly provide a wealth of knowledge on how a pathogen can manipulate host cells.

## IMPORTANCE

The pan-genome of the genus *Vibrio* is a potential reservoir of unidentified toxins that can provide insight into how members of this genus have successfully risen as emerging pathogens worldwide. We focused on *Vibrio proteolyticus*, a marine bacterium that was previously implicated in virulence toward marine animals, and characterized its interaction with eukaryotic cells. We found that this bacterium causes actin cytoskeleton rearrangements and leads to cell death. Using a proteomics approach, we identified a previously unstudied member of the leukocidin family of pore-forming toxins as the virulence factor responsible for the observed cytotoxicity in eukaryotic cells, as well as a plethora of additional putative virulence factors secreted by this bacterium. Our findings reveal a functional new clan of the leukocidin toxin superfamily and establish this pathogen as a reservoir of potential toxins that can be used for biomedical applications.

## INTRODUCTION

Reports on vibrios from around the world have been increasing in the past decade, with new pathogenic strains identified in America, Europe, Asia, and developing countries (1–4). Moreover, the spread of antibiotic resistance among vibrios has been reported (5, 6). Bacteria belonging to the genus *Vibrio* are known to share genetic material via horizontal gene transfer (7, 8). Many of the shared genes encode virulence factors that enhance bacterial fitness in the environment and mediate their interactions with competing bacteria, protist predators, and eukaryotic host cells during infection (7, 9, 10). All of the above emphasize the need to gain a more comprehensive understanding of virulence factors found in the *Vibrio* pan-genome.

Various virulence determinants have been identified and studied in vibrios. These include protein secretion systems and the toxins that they deliver. Some vibrios (e.g., *Vibrio vulnificus* and *Vibrio cholerae*) mostly rely on toxins secreted into the environment by the type I and type II secretion systems to mediate virulence activities (11–13). Many other vibrios (e.g., *Vibrio parahaemolyticus*) use type III secretion systems (T3SSs) to deliver effectors directly into the cytoplasm of eukaryotic host cells and manipulate cellular processes to their advantage (11, 14). More recently, type VI secretion systems (T6SSs) have been characterized in vibrios (9, 15–17). T6SSs, like T3SSs, are macromolecular machines used to deliver toxic effectors from the bacterium into neighboring cells in a contact-dependent manner (18, 19). Depending on the identity of the secreted effectors, T6SSs can mediate both virulence and antibacterial interactions (20, 21).

*Vibrio proteolyticus* is a Gram-negative marine bacterium originally isolated from the intestine of the wood borer *Limnoria tripunctata* (22). This organism has mainly been studied for the proteolytic enzymes that it secretes, which are used for industrial applications (23). While *V. proteolyticus* strains were found as part of the *Vibrio* consortium isolated from corals with yellow band disease (24) and were identified as pathogens of *Artemia* spp. (25), the identity of *V. proteolyticus* virulence factors has not been investigated. We hypothesized that *V. proteolyticus* possesses previously unidentified virulence determinants that can cause detrimental effects during interactions with eukaryotic cells. Here, we investigated the virulence potential of *V. proteolyticus* NBRC 13287 (ATCC 15338) and found that, when introduced to HeLa cells, this bacterium causes contact-independent, rapid morphological changes to the actin cytoskeleton, followed by cell lysis of the eukaryotic cells. Analysis of the *V. proteolyticus* secretome revealed several potential virulence factors, and upon further examination, we identified a secreted hemolysin belonging to the leukocidin superfamily of pore-forming toxins that was required for the *V. proteolyticus*-mediated cytotoxic effects. Moreover, we showed that this hemolysin is also required for *V. proteolyticus*-mediated cytotoxicity against macrophages. Bioinformatic analysis indicated that this secreted toxin represents a previously unstudied clan of the leukocidin pore-forming toxin superfamily and is found in several marine bacteria, including strains pathogenic to humans and marine animals.

## RESULTS

### *V. proteolyticus* induces actin cytoskeleton rearrangement and lysis in HeLa cells.

**FIG 1:**
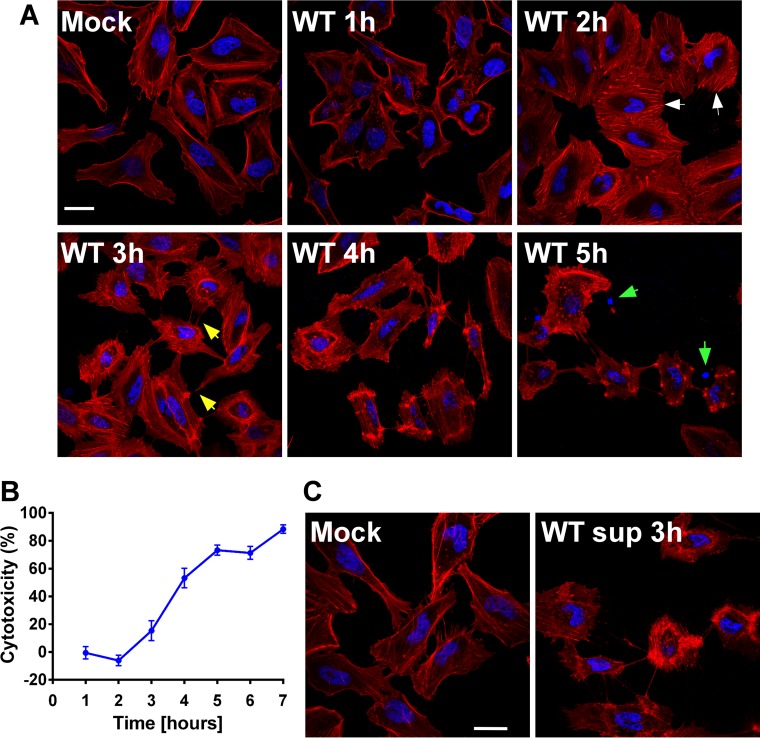
*V. proteolyticus* induces cytoskeletal rearrangements followed by lysis in HeLa cells. (A) Confocal micrograph of HeLa cells infected with wild-type (WT) *V. proteolyticus* for the indicated times and stained for F-actin and DNA using rhodamine-phalloidin (red) and Hoechst stain (blue), respectively. White arrows indicate actin stress fibers, yellow arrows indicate actin cables at retracting cell extremities, and green arrows indicate nuclei of lysed cells. Bar, 30 µm. (B) Measurements of LDH release from HeLa cells infected with *V. proteolyticus* to evaluate cytotoxicity shown as percentage of maximum lysis. Data shown as means ± standard deviations (*n* = 3). (C) Confocal micrograph of HeLa cells treated with concentrated *V. proteolyticus* supernatant (sup; 5-µg/ml final protein concentration) for 3 h. Cells were stained for F-actin and DNA using rhodamine-phalloidin (red) and Hoechst stain (blue), respectively. Bar, 30 µm.

To evaluate the outcome of *V. proteolyticus* interaction with eukaryotic cells, we first examined the effect of *V. proteolyticus* on a model eukaryotic cell line, human epithelial HeLa cells. To this end, we added *V. proteolyticus* wild-type bacteria, grown to logarithmic phase, to a HeLa cell culture at a multiplicity of infection (MOI) of 25 and monitored the actin cytoskeleton over the course of the infection. The infection of HeLa cells with *V. proteolyticus* induced dramatic rearrangement of the actin cytoskeleton within 2 h, with actin stress fibers appearing throughout the cell (Fig. 1A, white arrows). Within 3 h postinfection, HeLa cells began to shrink, leaving visible narrow actin cables at cell extremities (Fig. 1A, yellow arrows). Four hours postinfection, the number of HeLa cells that remained attached to the surface was decreased dramatically and the remaining cells appeared shrunken. After 5 h, only small numbers of shrunken cells, as well as free nuclei from lysed cells, were visible (Fig. 1A, green arrows).

To determine if the infection by *V. proteolyticus* was cytotoxic for HeLa cells, we monitored the release of lactate dehydrogenase (LDH) into the culture medium over the course of the infection. In agreement with the timing of the morphological changes seen in the actin cytoskeleton and the detachment of HeLa cells from the surface (Fig. 1A), LDH release was apparent starting at 3 h postinfection and increased over time (Fig. 1B). These results indicated that *V. proteolyticus* possesses a virulence determinant(s) that causes eukaryotic cell death.

### *V. proteolyticus*-mediated cell death is contact independent.

Bacterium-induced cell death can be caused by secreted toxins in either a contact-dependent or a contact-independent manner. To determine whether the *V. proteolyticus*-induced phenotypes observed in HeLa cells upon infection are contact dependent or not, we collected the proteins secreted by *V. proteolyticus* to the growth medium, added the concentrated supernatant to HeLa cell cultures (to a final protein concentration of 5 µg/ml), and monitored the effect on the actin cytoskeleton. As shown in Fig. 1C, addition of *V. proteolyticus* supernatant resulted in actin cytoskeleton rearrangements in HeLa cells as well as cell shrinking, similar to that observed during infection with the wild-type bacteria (compare with Fig. 1A). Based on these results, we concluded that *V. proteolyticus* secretes a cytotoxic toxin(s) that induces actin cytoskeleton rearrangements in a contact-independent manner.

### The *V. proteolyticus* secretome.

To identify the secreted proteins that mediate the cytotoxic effects of *V. proteolyticus*, we analyzed the *V. proteolyticus* secretome. To do so, we grew *V. proteolyticus* in liquid cultures and collected the media that contained the secreted proteins for analysis using label-free mass spectrometry (MS). Various classes of proteins were detected in the *V. proteolyticus* secretome, including flagellar proteins, nucleases, various peptidases and metalloproteases, and other hydrolytic enzymes (see Data Set S1 in the supplemental material). Among the potential virulence factors identified in the *V. proteolyticus* secretome are chitinases (GenBank sequence accession numbers GAD67278, GAD66187, GAD67897, GAD68832, and GAD68648), putative collagenases (accession numbers GAD66734 and GAD68603), neutral protease (accession number GAD68370), extracellular lipase (accession number GAD68208), a leukocidin hemolysin (accession number GAD67085), and a putative mucinase (accession number GAD66134). In addition, we identified several known secreted structural components of the T6SS, such as Hcp (accession numbers GAD65723, GAD66993, and GAD68053), VgrG (accession numbers GAD65726 and GAD66994), and PAAR-repeat-containing proteins (accession numbers GAD66641 and GAD67118) (26) (see Data Set S1), indicating that *V. proteolyticus*-encoded T6SSs are active under the examined growth conditions. Moreover, several predicted T6SS effectors that belong to the MIX effector class (27) were identified. These MIX effectors include putative antibacterial toxins (accession numbers GAD69221, GAD66988, GAD69368, and GAD67118) as well as putative virulence toxins such as those with accession numbers GAD68164 and GAD68163. The latter contains a virulence-mediating cytotoxic necrotizing factor 1 (CNF1) domain (27–29). It should be noted that we did not identify a T3SS in this bacterium, nor did we identify other common virulence factors, such as pili, in the secretome.

### *V. proteolyticus* T6SSs do not mediate HeLa cell lysis.

**FIG 2:**
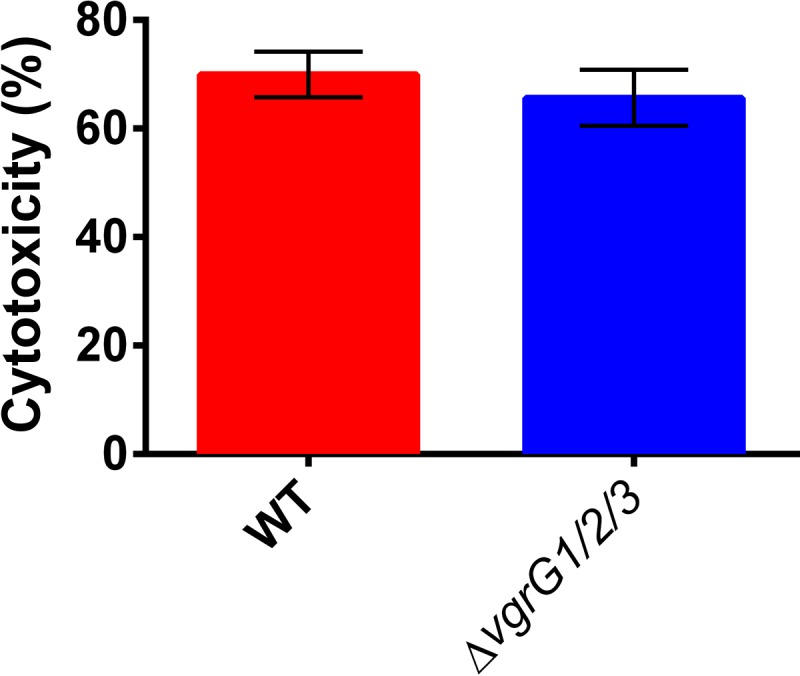
T6SSs are not required for *V. proteolyticus*-induced HeLa cell lysis. Measurements of LDH release from HeLa cells after 5 h of infection with wild-type (WT) *V. proteolyticus* or a strain in which all three *V. proteolyticus vgrG* genes were deleted (Δ*vgrG1/2/3*) to evaluate cytotoxicity shown as percentage of maximum lysis. Data shown as means ± standard deviations (*n* = 3).

Since we identified secreted T6SS MIX effectors with putative virulence activities in the *V. proteolyticus* secretome, we hypothesized that these T6SSs are responsible for the observed phenotypes in HeLa cells. Therefore, we tested whether the *V. proteolyticus* T6SSs were responsible for HeLa cell lysis. Because *V. proteolyticus* contains three T6SSs, we created a strain with deletions of all three genes encoding VgrG proteins (named the Δ*vgrG1/2/3* mutant), which are T6SS core components essential for T6SS activity (16, 19). We then used this mutant strain to infect HeLa cells and monitored cytotoxicity using the LDH release assay. As shown in Fig. 2, the cytotoxicity of the Δ*vgrG1/2/3* mutant after 5 h of infection was comparable to that of the wild-type *V. proteolyticus*, thus indicating that the cytotoxicity was not mediated by the *V. proteolyticus* T6SSs.

### A secreted hemolysin required for *V. proteolyticus*-mediated toxicity in HeLa cells.

**FIG 3:**
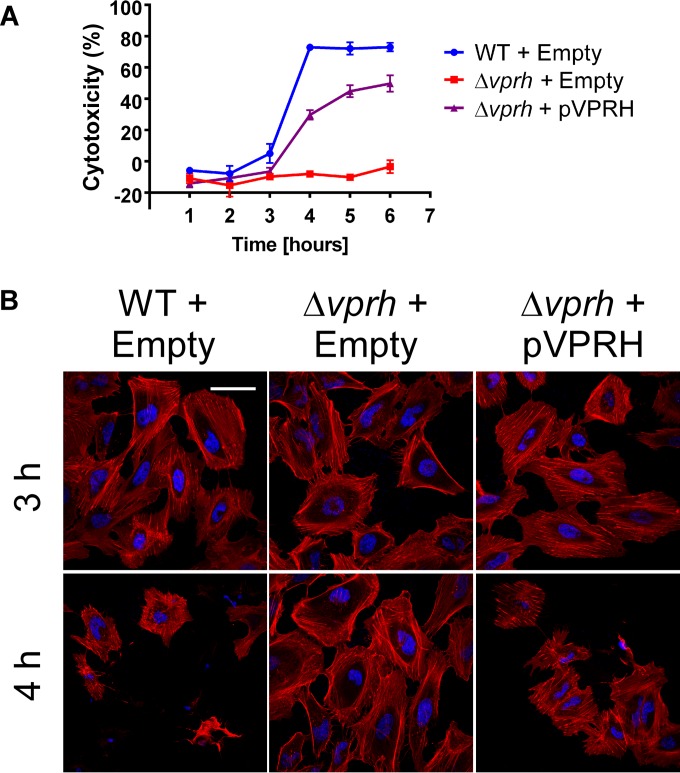
VPRH is required for *V. proteolyticus*-induced cytoskeletal rearrangements and lysis in HeLa cells. (A) Measurements of LDH release from HeLa cells infected with wild-type (WT) *V. proteolyticus* containing an empty vector (Empty) or *V. proteolyticus* in which *vprh* was deleted (Δ*vprh*) containing an empty vector or a vector for arabinose-inducible expression of VPRH (pVPRH). LDH release presented as percent cytotoxicity of maximum lysis. Data shown as means ± standard deviations (*n* = 3). (B) Confocal micrograph of HeLa cells infected with *V. proteolyticus* strains listed in panel A for the indicated time points and stained for F-actin and DNA using rhodamine-phalloidin (red) and Hoechst stain (blue), respectively. Bar, 30 µm.

Examining the *V. proteolyticus* secretome for virulence factors that mediate the observed cytotoxicity, we identified VPR01S_06_01020 (GenBank sequence accession number GAD67085), one of the most abundant proteins in the *V. proteolyticus* secretome (see Data Set S1 in the supplemental material), as the potential cytotoxin. The protein with accession number GAD67085 is a 305-amino-acid-long protein that contains an N-terminal cleavable signal peptide (SignalP 4.1 server [30]) and a domain that belongs to the leukocidin/hemolysin toxin superfamily (Pfam 07968). Here, we will refer to this protein as VPRH (*V. proteolyticus* hemolysin). Leukocidin-like hemolysins are predicted to be pore-forming toxins capable of lysing eukaryotic cells (31, 32). Pore-forming toxins have been previously shown to mediate cytoskeletal rearrangements and cause host cell death (32, 33). Therefore, we hypothesized that VPRH is the secreted toxin that mediates the observed actin cytoskeleton rearrangements and cell lysis in HeLa cells. To test this hypothesis, we generated a *vprh* deletion strain and proceeded to test the effect of *vprh* deletion on *V. proteolyticus*-mediated cytotoxicity. Remarkably, deletion of *vprh* resulted in complete loss of cytotoxicity (Fig. 3A). The *V. proteolyticus*-induced cytotoxicity was recovered by complementation of VPRH expressed exogenously from an arabinose-inducible vector (pVPRH) (Fig. 3A). Moreover, the actin cytoskeleton rearrangements and cell shrinking observed upon infection of HeLa cells with *V. proteolyticus* were completely absent when the *vprh* deletion strain was used for infection (Fig. 3B). These phenotypes were also recovered by exogenous expression of VPRH in the deletion strain (Fig. 3B). Notably, *vprh* deletion had no effect on bacterial growth, as shown in Fig. S1 in the supplemental material. Taken together, these results indicate that *V. proteolyticus* secretes VPRH, a leukocidin-like toxin that mediates actin cytoskeleton rearrangements followed by lysis of the infected host cell.

### VPRH is required for *V. proteolyticus*-mediated cytotoxicity in macrophages.

**FIG 4:**
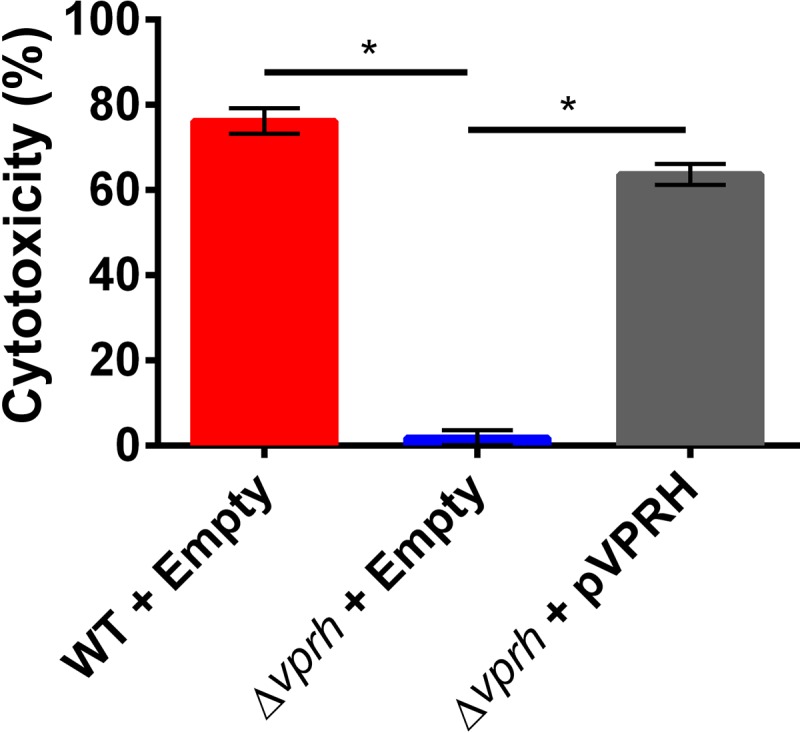
VPRH is required for *V. proteolyticus*-induced cell lysis in macrophages. Measurements of LDH release from RAW 264.7 cells after 5 h of infection with wild-type (WT) *V. proteolyticus* containing an empty vector (Empty) or *V. proteolyticus* in which *vprh* was deleted (Δ*vprh*) containing an empty vector or a vector for arabinose-inducible expression of VPRH (pVPRH). LDH release presented as percent cytotoxicity of maximum lysis. Data shown as means ± standard deviations (*n* = 3). Asterisks mark statistical significance between sample groups by an unpaired, two-tailed Student *t* test (*P* < 0.005).

As VPRH contains a predicted leukocidin domain, we next asked whether this toxin has cytotoxic effects on other cell types, such as leukocytes. To determine whether VPRH can mediate leukocyte cell lysis, we measured LDH release during *V. proteolyticus* infection of murine RAW 264.7 macrophages. Indeed, *V. proteolyticus* was cytotoxic to the macrophages as evidenced by LDH release (Fig. 4). As expected, deletion of *vprh* abolished the *V. proteolyticus*-mediated cytotoxic effect, which was recovered upon exogenous expression of VPRH in the deletion strain (Fig. 4). Thus, VPRH can target various eukaryotic cell types and induce cell lysis.

### VPRH represents an unstudied clan within the leukocidin superfamily.

**FIG 5:**
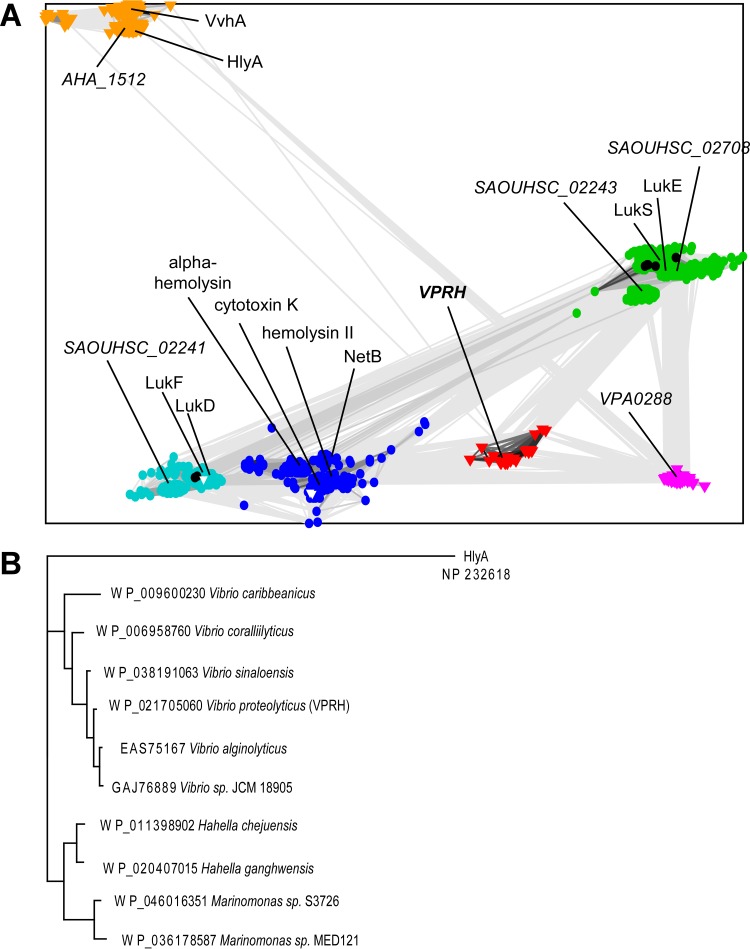
VPRH is a member of an unstudied clan of the leukocidin superfamily. (A) Homologs of VPRH were clustered in two dimensions according to sequence similarity, with nodes representing each sequence and connecting lines representing distance. Nodes from Gram-positive bacteria are depicted as circles, and nodes from Gram-negative bacteria are depicted as triangles, and both are colored according to clusters: VPRH-like (red), HlyA-like (orange), VPA0288-like (magenta), LukE/S-like (green), cytotoxin K-like (blue), and LukD/F-like (cyan). Three sequences from Gram-negative bacteria are annotated as partial sequences and cluster with the Gram positives (white triangles) and likely represent either horizontally transferred genes or sequencing errors. Viral sequences are black. Sequences from several complete genomes (*V. proteolyticus*, *V. cholerae*, *V. parahaemolyticus*, *Bacillus cereus*, *S. aureus*, and *Aeromonas hydrophila*) are labeled according to the protein name (for experimentally described sequences) or to the gene symbol (unknown function). (B) A maximum likelihood tree corresponding to the conserved leukocidin-like toxin domain from representative VPRH family sequences rooted with the *V. cholerae* HlyA sequence outgroup.

VPRH homologs include representatives from other Gram-negative *Proteobacteria* (*Betaproteobacteria* and *Gammaproteobacteria*) and from Gram-positive *Firmicutes* (*Bacillales*, *Clostridiales*, and *Lactobacillales*), for which several different three-dimensional structures are available. The superfamily is defined by a common core leukocidin-like toxin domain comprised of an immunoglobulin-like β-sandwich cap (34, 35) with a pore-forming β-strand insert that adopts alternate conformations in the soluble and oligomeric pore-forming states (36). The toxins also include a rim subdomain (35) formed by three to four different loops that extend from the same side of the immunoglobulin sandwich (see Fig. S2 in the supplemental material). As shown in Fig. 5A, the leukocidin-like toxin sequences tend to cluster according to domain organization (in the distant *VCA0219* HlyA cluster) as well as variable regions of the leukocidin-like toxin that map to the rim subdomain. A phylogenetic analysis based on the common core regions of the leukocidin-like toxin separates the sequences into Gram-positive and Gram-negative ones (Fig. 5A, circles and triangles, respectively). The Gram-positive sequences, which are sometimes associated with phage, tend to exist in multiple copies in the genome that likely resulted from duplications arising after speciation.

Members of the leukocidin superfamily have been previously reported to mediate critical virulence functions in clinically important bacterial pathogens (31, 37–39). However, members of two leukocidin superfamily clans, represented by VPRH (Fig. 5A, red triangles) and *V. parahaemolyticus* VPA0288 (Fig. 5A, magenta triangles), have not been previously identified or characterized. Our results indicate that the members of the VPRH clan, which are present in several species of marine bacteria such as *Vibrio*, *Hahella*, and *Marinomonas* spp. (Fig. 5B; see also Fig. S3 in the supplemental material), may be functional cytotoxins capable of causing cell death in multiple eukaryotic cell types.

## DISCUSSION

Bacteria that are not known to be pathogenic to humans carry putative virulence determinants. In this work, we set out to identify new virulence traits in the *Vibrio* pan-genome by investigating the interaction of the marine bacterium *V. proteolyticus* with eukaryotic cells. Using a proteomics approach, we determined the *V. proteolyticus* secretome in pursuit of toxins that mediated the cytotoxic effects that we observed when infecting HeLa cells with *V. proteolyticus*.

When examining the *V. proteolyticus* secretome, we discovered that a leukocidin-like hemolysin, which we named VPRH, is the secreted toxin responsible for the cytotoxic effects against both HeLa cells and murine macrophages. Interestingly, members of the leukocidin superfamily are found in many human pathogens and have been shown to play key roles in their pathogenicity (31, 32, 35, 38). For example, leukocidin toxins found in Gram-positive bacterial pathogens, such as *Staphylococcus aureus*, were shown to play unique roles in promoting disease (31). In addition, Gram-negative bacterial pathogens also employ leukocidin-like toxins that promote virulence, such as the *V. vulnificus* VvhA toxin (38). VPRH represents a previously uncharacterized clan of the leukocidin superfamily (Fig. 5A). Members of the VPRH-leukocidin clan are present in *Vibrio* spp. and other marine bacteria with mostly ecological and environmental importance (Fig. 5B). Of note are the coral pathogen *Vibrio coralliilyticus* (40), the shrimp pathogen *Vibrio sinaloensis* (41), and *Hahella chejuensis*, which produce prodigiosin, which is an algicidal compound used to control harmful algal blooms (42). In addition, close homologs of VPRH are found in *Vibrio alginolyticus*, which is a pathogen of marine animals and also known to be a human pathogen (43–45). Further studies will be required to determine the biophysical properties of VPRH and its host cell receptor(s).

Another previously uncharacterized clan of the leukocidin superfamily appears to be made up of close homologs of VPA0288 from *V. parahaemolyticus* RIMD 2210633 (Fig. 5A), which are found exclusively in *Vibrio* (i.e., *V. parahaemolyticus*, *Vibrio harveyi*, *Vibrio* sp. S234-5, and *Vibrio alginolyticus*) and *Photobacterium* spp. In contrast to VPRH, VPA0288 does not seem to actively mediate cytotoxic effects against HeLa cells, as it was not previously reported as a cytotoxic factor during HeLa cell infections. VPA0288 was previously shown to be expressed and secreted from *V. parahaemolyticus*, albeit at very low levels (27), and yet, past studies have shown that the only virulence factors that mediate HeLa cell lysis during infections of cell cultures are the thermostable direct hemolysin (TDH)/TDH-related hemolysin (TRH) and the T3SSs encoded by this pathogen (46–48). These differences in apparent activity between VPRH and VPA0288 may be attributed to differences in expression or secretion levels of the toxins under the tested conditions or due to the variable leukocidin rim subdomains that perhaps mediate binding specificity to different receptors on the target cell and thus target a different subset of host cells. Interestingly, members of the third cluster of the leukocidin superfamily that are found in Gram-negative bacteria (such as the *V. cholerae* HlyA and the *V. vulnificus* VvhA [Fig. 5A, orange triangles]) contain additional C-terminal lectin domains that contribute to their host recognition (49).

Many other potential virulence factors were identified in the secretome of *V. proteolyticus*, including various proteases, chitinases, collagenases, and a putative mucinase. In addition, we found that several structural T6SS components were secreted by *V. proteolyticus*, as well as predicted T6SS MIX effectors (27) with virulence, antibacterial, and unknown toxin domains. Four of the identified MIX effectors belong to the MIX V clan (GenBank sequence accession numbers GAD68163, GAD68164, GAD69368, and GAD69221), which we recently reported are mobile toxins shared between marine bacteria via horizontal gene transfer (9). Thus, it is possible that MIX effectors with virulence toxin domains, such as the CNF1 domain-containing MIX effector VPR01S_11_01570 (accession number GAD68163), can find their way into other pathogenic *Vibrio* species with pandemic potential (e.g., *V. parahaemolyticus*, which also harbors a T6SS that can deliver MIX effectors [9, 27]) via horizontal gene transfer, adding to their virulence traits and presenting new pathogenic potential. Notably, horizontal acquisition of new virulence traits was recently reported in *V. parahaemolyticus*, leading to its emergence as a worldwide pathogen decimating cultured shrimp farms (10). Whereas the *V. proteolyticus* T6SSs were not responsible for the cytotoxic effects that we witnessed in HeLa cells, further studies are required to determine the virulence activities of the *V. proteolyticus* T6SSs and their effector repertoire.

The presence of various potential virulence determinants in the *V. proteolyticus* secretome, together with the identification of the functional cytotoxic VPRH, points to *V. proteolyticus* as a reservoir of potential virulence factors. It will be interesting to determine how these factors contribute to pathogenesis of this bacterium as an individual clone, as well as part of a consortium (24). Our results suggest that the *Vibrio* pan-genome contains unknown and underappreciated virulence factors that deserve further investigation. These virulence factors have the potential to spread to other pathogenic members of the *Vibrio* genus, giving rise to potentially new pathogenic strains. Moreover, such novel toxins can be used for biomedical and biocontrol applications (50), especially if they present specificity for target cell lines, as could be the case for VPRH identified in this work.

## MATERIALS AND METHODS

### Bacterial strains and cell culture.

*Vibrio proteolyticus* strain NBRC 13287 (ATCC 15338) and its derivative strains were routinely grown in marine Luria-Bertani (MLB) broth (Luria-Bertani broth supplemented with NaCl to a final concentration of 3%) at 30°C. When necessary, medium was supplemented with 200 µg/ml kanamycin or 25 µg/ml chloramphenicol. To induce expression of genes from a plasmid, 0.1% (wt/vol) l-arabinose was included in the medium. HeLa cells (ATCC) and RAW 264.7 murine macrophages (ATCC) were cultured in high-glucose Dulbecco’s modified Eagle’s medium (DMEM [Gibco]) supplemented with 10% (vol/vol) fetal bovine serum, 1% (vol/vol) penicillin-streptomycin–glutamine, and 1% (vol/vol) sodium pyruvate and kept at 5% CO_2_ at 37°C.

### Plasmid construction.

A derivative of the pBAD/*Myc*-His vector (Invitrogen), in which the ampicillin resistance cassette was replaced with a kanamycin resistance cassette, was used to generate an N-terminal *c-myc* tag vector by inserting a sequence encoding a *c-myc* tag between the NcoI and XhoI sites of the multiple cloning site (MCS). The primers used were 5′ CATGGAACAGAAACTGATTTCCGAAGAGGATCTGC 3′ and 5′ TCGAGCAGATCCTCTTCTGAGATGAGTTTTTGTTC 3′. The resulting plasmid was named pMBAD. For arabinose-inducible expression of *vpr01s_06_01020* (GenBank sequence accession number GAD67085), the coding sequence of the gene was amplified from a genomic DNA preparation of *V. proteolyticus* using primers 5′ CACCCTCGAGATGAAAAAACGGCACCTTTC 3′ and 5′ CAACGGTACCTTAATGGTAGAGCGCGTCG 3′ and subsequently cloned between the XhoI and KpnI sites of the pMBAD MCS with the inclusion of the stop codon at the 3′ end, to generate pVPRH.

### Construction of deletion strains.

For in-frame deletions of *vprh* and T6SS genes *vgrG1* (GenBank sequence accession number GAD66994), *vgrG2* (accession number GAD67611), and v*grG3* (accession number GAD65726), 1-kb sequences directly upstream and downstream of each gene were cloned into pDM4, a Cm^r^ OriR6K suicide plasmid (51). These pDM4 constructs were inserted into *V. proteolyticus* via conjugation by S17-1(λ*pir*) *Escherichia coli*. Transconjugants were selected for on MLB agar containing chloramphenicol. The resulting transconjugants were plated onto minimal marine medium agar containing 15% (wt/vol) sucrose for counterselection and loss of the *sacB*-containing pDM4. Deletions were confirmed by PCR.

### Bacterial growth assay.

Overnight-grown cultures of *V. proteolyticus* were normalized to an optical density at 600 nm (OD_600_) of 0.1 in 25 ml MLB in triplicates and grown at 30°C with agitation for 6 h. Growth was assessed by OD_600_ measurements at indicated time points. Experiments were performed at least twice with similar results. Results of a representative experiment are shown.

### Tissue culture infection assay.

HeLa cells were seeded at 7 × 10^4^ cells/ml in 6-well plates containing UV-pretreated 22- by 22-mm glass coverslips. Overnight cultures of *V. proteolyticus* were normalized to an OD_600_ of 0.1 in 5 ml MLB and grown for 2 h with agitation at 30°C. When necessary, 200 µg/ml kanamycin and 0.1% (wt/vol) l-arabinose were added to maintain plasmids and induce expression. OD_600_ measurements of the cultures were taken and used to calculate the volume needed to make solutions of DMEM without antibiotics (termed infection media) containing a multiplicity of infection (MOI) of 25 for each bacterial strain. The infection solutions included 200 µg/ml kanamycin and 0.1% (wt/vol) l-arabinose when necessary. HeLa cells were washed twice with 1 ml of unsupplemented DMEM, and then 2 ml infection medium with bacteria was added to each well. The plates were centrifuged at 200 × *g* for 5 min to synchronize infection before incubation at 37°C with 5% CO_2_ for the indicated duration.

For testing the effect of proteins secreted by *V. proteolyticus* on HeLa cells, an overnight culture was normalized to an OD_600_ of 1.0 in 5 ml unsupplemented DMEM and incubated at 37°C for 3 h with agitation. The culture was centrifuged at 3,220 × *g* at 4°C for 10 min to pellet the bacteria. The culture supernatant containing the secreted proteins was filter sterilized using an 0.22-μm filter. To concentrate the secreted proteins, 4 ml of the filtered supernatant was added to an Ultracel-10K centrifugal filter (Amicon) and centrifuged at 3,220 × *g* at 4°C to a volume of 150 µl. Protein concentration was determined using the Bradford assay. The protein concentrate was added to HeLa cells to produce a final concentration of 5 µg/ml protein in 2 ml infection medium, and cells were incubated at 37°C with 5% CO_2_ for 3 h. Experiments were performed at least twice with similar results. Results of a representative experiment are shown.

### LDH cytotoxicity assay.

HeLa cells were seeded at 7.5 × 10^4^ cells/ml in 24-well plates. For RAW 264.7 macrophages, cells were seeded at 5 × 10^5^ cells/ml. Overnight cultures of *V. proteolyticus* were normalized to an OD_600_ of 0.1 in 5 ml MLB and grown for 2 h at 30°C with agitation. When necessary, 200 µg/ml kanamycin and 0.1% (wt/vol) l-arabinose were added to maintain plasmids and induce protein expression. Infection solutions with an MOI of 25 were prepared in DMEM free of supplements and phenol red (clear DMEM). Cells were washed twice with 1 ml of clear DMEM, and then 1 ml infection solution was added in triplicate for each strain. Duplicates of high and low control wells were also included for each time point. Further methodological details on LDH release measurements can be found in Text S1 in the supplemental material. Experiments were performed at least twice with similar results. Results of a representative experiment are shown.

### Microscopy.

Slides were imaged using a Zeiss LSM 710 confocal microscope, and images were converted using ImageJ (NIH). Further methodological details on slide preparations can be found in Text S1 in the supplemental material.

### Mass spectrometry.

For analysis of its secretome, overnight cultures of *V. proteolyticus* were normalized to an OD_600_ of 0.18 in 50 ml MLB medium in triplicates and grown for 5 h at 30°C. Media were collected, and proteins were precipitated as previously described using deoxycholate and trichloroacetic acid (52). Protein samples were run 10 mm into the top of an SDS-PAGE gel, stained with Coomassie blue, and excised. Further methodological details on sample preparation and analysis can be found in Text S1 in the supplemental material.

### Sequence analysis.

The *V. proteolyticus* sequence of VPRH was queried against the nonredundant protein sequence (nr) database using PSI-BLAST (53) (E value cutoff of 0.005) for 3 iterations to identify homologs. Further methodological details on alignments and clustering of sequences can be found in Text S1 in the supplemental material.

### Accession number(s).

The raw MS data files and the corresponding peak lists were uploaded to the MassIVE data set repository under MassIVE identification number MSV000079754 (http://massive.ucsd.edu/ProteoSAFe/status.jsp?task=d7546937dfa443f8949c94fac60fa2ec).

## SUPPLEMENTAL MATERIAL

Figure S1  Deletion of *vprh* does not affect *V. proteolyticus* growth. Growth of *V. proteolyticus* strains in MLB at 30°C measured as absorbance optical density at 600 nm (OD_600_). Data are means ± standard deviations (*n* = 3). WT, wild type. Download Figure S1, TIF file, 0.1 MB

Figure S2  Representative structures of leukocidin-like toxins. Structures are represented as cartoons and colored according to subdomains: immunoglobulin-like sandwich of the leukocidin-like domain (cyan), conformation changing pore-forming insert (green), and rim subdomain (gray, wheat, pink, and red). (A) LukD represented in the soluble conformation (PDB ID 4q7g). (B) HlyA represented in the soluble conformation (PDB ID 1xez), with an N-terminal pro domain (blue), a C-terminal ricin-like domain (yellow), and a C-terminal jacalin-like domain. (C and D) A single chain (C) and the assembled pore (D; additional chains in white) of alpha-hemolysin in the pore-forming conformation (PDB ID 7ahl). Download Figure S2, TIF file, 2.1 MB

Figure S3  Multiple sequence alignment of VPRH-clan members of the leukocidin superfamily. Close VPRH family sequence representatives (labeled to the left with NCBI accession numbers) are aligned with several leukocidin-like toxin structure sequences (top four, labeled to the left with PDB ID). Conserved leukocidin-like toxin sequence positions are highlighted according to conservation: mainly hydrophobic (yellow), mainly small (gray), mainly aromatic (dark yellow), invariant residues (black) or conserved polar residues (black, with conserved alternate amino acids colored yellow). The conserved helical (H) and strand (E) secondary structure (SS) elements of leukocidin-like toxin structures are indicated above the alignment and are highlighted according to subdomain: toxin immunoglobulin-like core (cyan), pore-forming hairpin (green), and rim loops (gray, pink, red, and wheat) also labeled above the SS. Invariant VPRH family residues in the rim subdomain are highlighted in the same color as the containing loop. Download Figure S3, TIF file, 0.6 MB

Data Set S1  Mass spectrometry results for *V. proteolyticus* secretome. Download Data Set S1, XLSX file, 0.1 MB

Text S1  Supplemental materials and methods. Download Text S1, DOCX file, 0.1 MB
