# Supplementary figures and images for: Proteomics Analysis Reveals Previously Uncharacterized Virulence Factors in Vibrio proteolyticus
Source: mBio. 2016 Jul 26;7(4):e01077-16. doi: 10.1128/mBio.01077-16 (PMC4981721; doi:10.1128/mBio.01077-16)

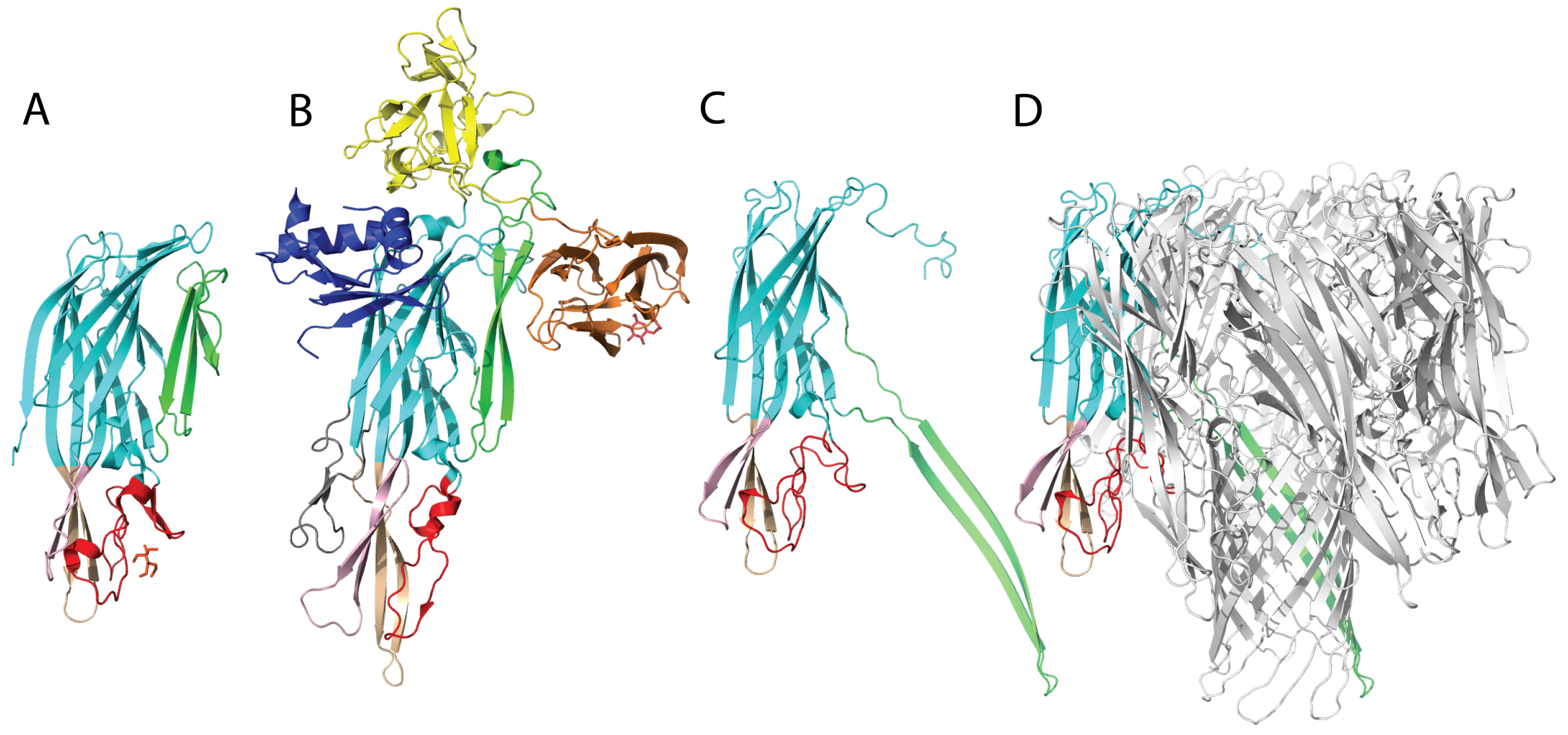

Supplement: Figure S2 — Representative structures of leukocidin-like toxins. Structures are represented as cartoons and colored according to subdomains: immunoglobulin-like sandwich of the leukocidin-like domain (cyan), conformation changing pore-forming insert (green), and rim subdomain (gray, wheat, pink, and red). (A) LukD represented in the soluble conformation (PDB ID 4q7g). (B) HlyA represented in the soluble conformation (PDB ID 1xez), with an N-terminal pro domain (blue), a C-terminal ricin-like domain (yellow), and a C-terminal jacalin-like domain. (C and D) A single chain (C) and the assembled pore (D; additional chains in white) of alpha-hemolysin in the pore-forming conformation (PDB ID 7ahl). Download [file mbo004162919sf2.tif]

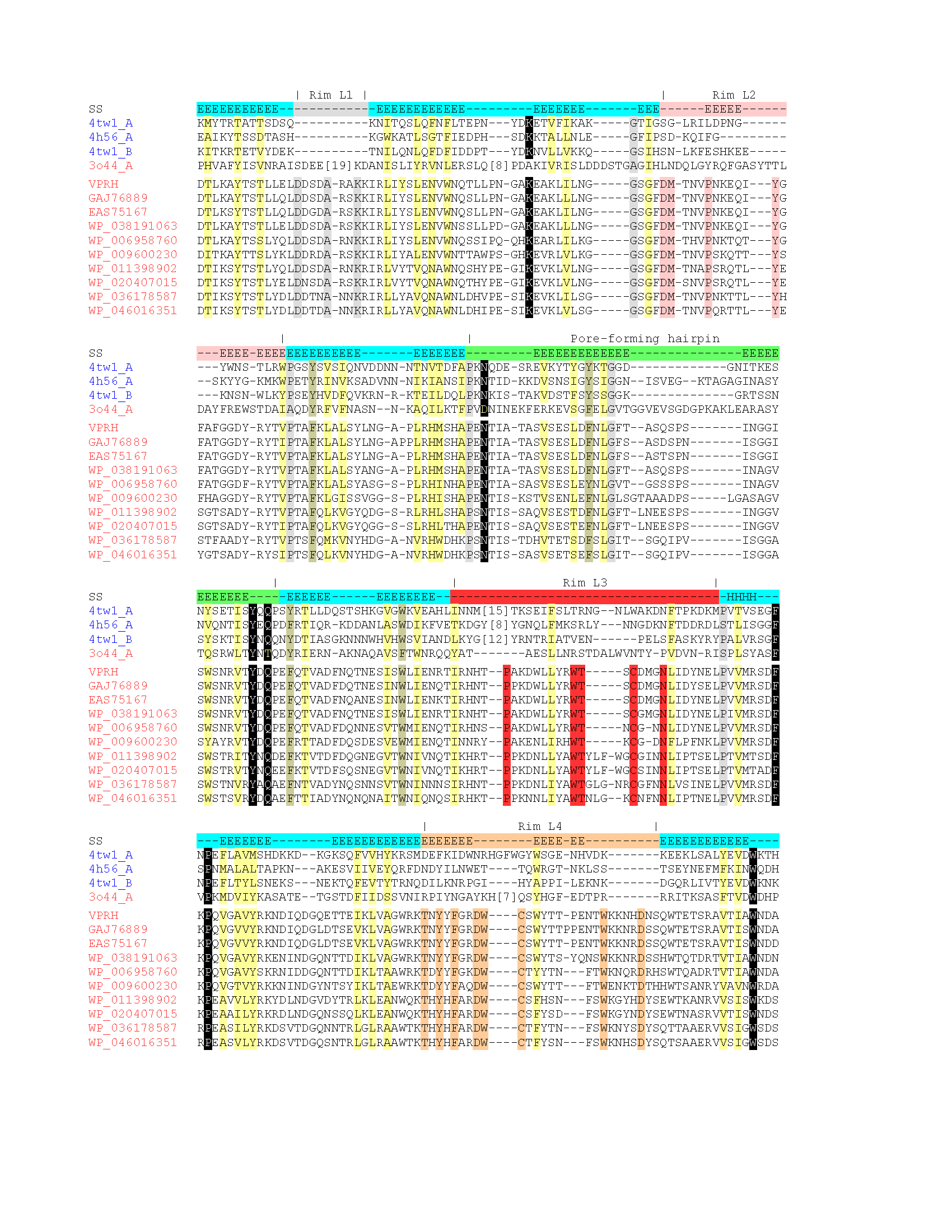

Supplement: Figure S3 — Multiple sequence alignment of VPRH-clan members of the leukocidin superfamily. Close VPRH family sequence representatives (labeled to the left with NCBI accession numbers) are aligned with several leukocidin-like toxin structure sequences (top four, labeled to the left with PDB ID). Conserved leukocidin-like toxin sequence positions are highlighted according to conservation: mainly hydrophobic (yellow), mainly small (gray), mainly aromatic (dark yellow), invariant residues (black) or conserved polar residues (black, with conserved alternate amino acids colored yellow). The conserved helical (H) and strand (E) secondary structure (SS) elements of leukocidin-like toxin structures are indicated above the alignment and are highlighted according to subdomain: toxin immunoglobulin-like core (cyan), pore-forming hairpin (green), and rim loops (gray, pink, red, and wheat) also labeled above the SS. Invariant VPRH family residues in the rim subdomain are highlighted in the same color as the containing loop. Download [file mbo004162919sf3.tif]
