# Supplementary material for: Proteomics Analysis Reveals Previously Uncharacterized Virulence Factors in Vibrio proteolyticus
Source: mBio. 2016 Jul 26;7(4):e01077-16. doi: 10.1128/mBio.01077-16 (PMC4981721; doi:10.1128/mBio.01077-16)
Supplement: Text S1 — Supplemental materials and methods. Download [file mbo004162919s1.docx]

Supplemental Text S1

**Proteomics analysis reveals previously uncharacterized virulence factors in *Vibrio proteolyticus***

Ann Ray^a,b^, Lisa N. Kinch^b^, Marcela de Souza Santos^a^, Nick V. Grishin^b,c,d^,

Kim Orth^a,b,c,#^, and Dor Salomon^a,#^

Department of Molecular Biology^a^, Howard Hughes Medical Institute^b^, Department of Biochemistry^c^, Department of Biophysics^d^, University of Texas Southwestern Medical Center, Dallas, Texas, USA

**SUPPLEMENTARY MATERIALS AND METHODS**

**LDH cytotoxicity assay.** Following infections, plates were centrifuged at 200 x *g* for 5 minutes to synchronize infection before being placed at 37°C with 5% CO_2_ to incubate. Fifteen minutes before each time point, the media from the high control wells was replaced with 1 mL of 1% of triton X-100 in 1x phosphate-buffered saline (PBS, pH 7.4). At each time point, three technical replicates of 200 μL were taken from the media of each well and pipetted into a 96-well plate. This plate was then centrifuged at 200 x *g* for 5 minutes to pellet any dead cells floating in the media. After centrifugation, samples of 100 μL were taken from each replicate into a fresh 96-well plate and stored at 4°C for a maximum of 24 hours. The Clontech LDH Cytotoxicity Detection Kit was used to quantify LDH release according to the manufacturer protocol.

**Microscopy.** Following infection assays, cells were washed once with PBS (pH 7.4) and fixed to the coverslips by treating with 1 mL of 3.2% paraformaldehyde solution at 4°C overnight. Fixed cells were treated the following day with 0.1% triton X-100 for 10 minutes to permeabilize membranes. The cells were washed with 1 mL PBS and were subsequently stained with 200 μL of a dye solution for 10 minutes, covered. The dye solution contained a 1:1000 dilution of 1mg/mL Hoechst 33342 (Molecular Probes) and a 1:100 dilution of 200 U/mL rhodamine-phalloidin (Molecular Probes) to stain nuclei and F-actin, respectively. Cells were treated three times to a 5-minute wash with 1 mL PBS to reduce background staining. After the final wash, the coverslips were mounted onto glass slides using a drop of ProLong Gold antifade mounting solution (Molecular Probes), and sealed after sitting at room-temperature for several hours.

**Mass spectrometry.** Samples underwent solid-phase extraction cleanup with Oasis HLB plates (Waters) and the resulting samples were analyzed by LC/MS/MS using a Thermo Fusion Lumos mass spectrometer (Thermo) coupled to an Ultimate 3000 RSLC-Nano liquid chromatography systems (Dionex). Samples were injected onto a 75 μm i.d., 50-cm long, 2µ, C18 Easy Spray column (Thermo) and eluted with a gradient from 0-28% buffer B over 60 min. Buffer A contained 2% (v/v) acetonitrile (ACN) and 0.1% formic acid in water, and buffer B contained 80% (v/v) ACN, 10% (v/v) trifluoroethanol, and 0.08% formic acid in water. The mass spectrometer acquired up to 10 MS/MS spectra for each full spectrum acquired, using higher-energy collisional dissociation (HCD) for peptide fragmentation.

Raw MS data files were converted to a peak list format and analyzed using the central proteomics facilities pipeline (CPFP), version 2.0.3 ([1](#_ENREF_1), [2](#_ENREF_2)). Peptide identification was performed using the X!Tandem ([3](#_ENREF_3)) and open MS search algorithm (OMSSA) ([4](#_ENREF_4)) search engines against the *Vibrio proteolyticus* NBRC 13287 protein database from NCBI (http://www.ncbi.nlm.nih.gov/). Fragment and precursor tolerances of 20 ppm and 0.5 Da were specified, and three missed cleavages were allowed. Carbamidomethylation of Cys was set as a fixed modification, with oxidation of Met set as a variable modification. Label-free quantitation of proteins across samples was performed using SINQ normalized spectral index software ([5](#_ENREF_5)). Protein identifications were filtered to an estimated 1% protein false discovery rate (FDR) using the concatenated target-decoy method ([6](#_ENREF_6)), and proteins needed to be present in all three samples to be considered. Additional requirements of two unique peptide sequences per protein and three spectral counts per protein per replicate were imposed, resulting in a final protein FDR <1%. The raw MS data files and the corresponding peak lists were uploaded to the MassIVE dataset repository under MassIVE ID: MSV000079754 (http://massive.ucsd.edu/ProteoSAFe/status.jsp?task=d7546937dfa443f8949c94fac60fa2ec).

**Sequence analysis.** Hits (1792 complete sequences including 30 structures) were collected and clustered using CLANS ([7](#_ENREF_7)) (*P*-value cutoff 0.01). Several outlier sequences were removed (WP_008709645.1, WP_061619592.1, ACY14651.1, KGW18030.1, KGX17281.1, KGX23826.1, WP_045118831.1) from the clustering. Representative sequences from the VPRH cluster were aligned with PROMALS-3D ([8](#_ENREF_8)), using several structures solved in the pore-forming conformation as guides (4tw1 chain A, 4tw1 chain B, 3o44, and 4h56). Distances for the VPRH cluster representative sequences with the HlyA sequence as an outgroup were calculated using the Molphy package ([9](#_ENREF_9)) maximum likelihood inference of protein phylogeny (protml) with the JTT-F model. An initial neighbor joining tree was produced from the same alignment using neighbor joining ([10](#_ENREF_10)) with the njdist program, and this tree was submitted to local rearrangement search strategy mode of protml to generate the final tree rooted with the HlyA outgroup.

**SUPPLEMEMTAL REFERENCES**

1. **Trudgian DC, Mirzaei H.** 2012. Cloud CPFP: a shotgun proteomics data analysis pipeline using cloud and high performance computing. J Proteome Res **11:**6282-6290.

2. **Trudgian DC, Thomas B, McGowan SJ, Kessler BM, Salek M, Acuto O.** 2010. CPFP: a central proteomics facilities pipeline. Bioinformatics **26:**1131-1132.

3. **Craig R, Beavis RC.** 2004. TANDEM: matching proteins with tandem mass spectra. Bioinformatics **20:**1466-1467.

4. **Geer LY, Markey SP, Kowalak JA, Wagner L, Xu M, Maynard DM, Yang X, Shi W, Bryant SH.** 2004. Open mass spectrometry search algorithm. J Proteome Res **3:**958-964.

5. **Trudgian DC, Ridlova G, Fischer R, Mackeen MM, Ternette N, Acuto O, Kessler BM, Thomas B.** 2011. Comparative evaluation of label‐free SINQ normalized spectral index quantitation in the central proteomics facilities pipeline. Proteomics **11:**2790-2797.

6. **Elias JE, Gygi SP.** 2007. Target-decoy search strategy for increased confidence in large-scale protein identifications by mass spectrometry. Nat Methods **4:**207-214.

7. **Frickey T, Lupas A.** 2004. CLANS: a Java application for visualizing protein families based on pairwise similarity. Bioinformatics **20:**3702-3704.

8. **Pei J, Tang M, Grishin NV.** 2008. PROMALS3D web server for accurate multiple protein sequence and structure alignments. Nucleic Acids Res **36:**W30-34.

9. **Adachi J, Hasegawa M.** 1992. Molphy: Programs for molecular phylogenetics based on maximum likelihood. Institute of Statistical Mathematics, Tokyo.

10. **Saitou N, Nei M.** 1987. The neighbor-joining method: a new method for reconstructing phylogenetic trees. Mol Biol Evol **4:**406-425.

**SUPPLEMENTAL FIGURE LEGENDS**

**Fig. S1. Deletion of *vprh* does not affect *Vpr* growth.** Growth of *Vpr* strains in MLB at 30°C measured as absorbance optical density at 600 nm (OD_600_). Data are means ± SD (n=3). WT = wild-type.

**Fig. S2. Representative structures of leukocidin-like toxins.** Structures are represented as cartoons and colored according to subdomains: immunoglobulin-like sandwich of the leukocidin-like domain (cyan), conformation changing pore-forming insert (green), and rim subdomain (grey, wheat, pink, and red). (**A**) LukD represented in the soluble conformation [PDB ID 4q7g]. (**B**) HlyA represented in the soluble conformation [PDB ID 1xez], with an N-terminal pro domain (blue), a C-terminal ricin-like domain (yellow), and a C-terminal jacalin-like domain. (**C-D**) a single chain (C) the assembled pore (D; additional chains in white) of alpha-hemolysin in the pore-forming conformation [PDB ID 7ahl].

**Fig. S3. Multiple sequence alignment of VPRH-clan members of the leukocidin superfamily.** Close VPRH family sequence representatives (labeled to the left with NCBI accession) are aligned with several leukocidin-like toxin structure sequences (top four, labeled to the left with PDB ID). Conserved leukocidin-like toxin sequence positions are highlighted according to conservation: mainly hydrophobic (yellow), mainly small (gray), mainly aromatic (dark yellow), invariant residues (black) or conserved polar residues (black, with conserved alternate amino acids colored yellow). The conserved helical (H) and strand (E) secondary structure (SS) elements of leukocidin-like toxin structures is indicated above the alignment and is highlighted according to subdomain: toxin immunoglobulin-like core (cyan), pore-forming hairpin (green), and rim loops (gray, pink, red, and wheat) also labeled above the SS. Invariant VPRH family residues in the Rim subdomain are highlighted in the same color as the containing loop.
